# A Network Model of Local Field Potential Activity in Essential Tremor and the Impact of Deep Brain Stimulation

**DOI:** 10.1371/journal.pcbi.1005326

**Published:** 2017-01-09

**Authors:** Nada Yousif, Michael Mace, Nicola Pavese, Roman Borisyuk, Dipankar Nandi, Peter Bain

**Affiliations:** 1 Division of Brain Sciences, Imperial College London, London, United Kingdom; 2 School of Engineering and Technology, University of Hertfordshire, Hatfield, United Kingdom; 3 Department of Bioengineering, Imperial College London, London, United Kingdom; 4 School of Computing and Mathematics, University of Plymouth, Plymouth, United Kingdom; 5 Institute of Mathematical Problems of Biology of RAS, The Branch of Keldysh Institute of Applied Mathematics of Russian Academy of Sciences, Moscow, Russia; École Normale Supérieure, College de France, CNRS, FRANCE

## Abstract

Essential tremor (ET), a movement disorder characterised by an uncontrollable shaking of the affected body part, is often professed to be the most common movement disorder, affecting up to one percent of adults over 40 years of age. The precise cause of ET is unknown, however pathological oscillations of a network of a number of brain regions are implicated in leading to the disorder. Deep brain stimulation (DBS) is a clinical therapy used to alleviate the symptoms of a number of movement disorders. DBS involves the surgical implantation of electrodes into specific nuclei in the brain. For ET the targeted region is the ventralis intermedius (Vim) nucleus of the thalamus. Though DBS is effective for treating ET, the mechanism through which the therapeutic effect is obtained is not understood. To elucidate the mechanism underlying the pathological network activity and the effect of DBS on such activity, we take a computational modelling approach combined with electrophysiological data. The pathological brain activity was recorded intra-operatively via implanted DBS electrodes, whilst simultaneously recording muscle activity of the affected limbs. We modelled the network hypothesised to underlie ET using the Wilson-Cowan approach. The modelled network exhibited oscillatory behaviour within the tremor frequency range, as did our electrophysiological data. By applying a DBS-like input we suppressed these oscillations. This study shows that the dynamics of the ET network support oscillations at the tremor frequency and the application of a DBS-like input disrupts this activity, which could be one mechanism underlying the therapeutic benefit.

## Author Summary

Essential tremor (ET) is acknowledged to be the most common movement disorder affecting 1% of the population. Although the underlying mechanisms remain elusive, the thalamus, cortex and cerebellum are implicated in the underlying pathology. More recently, it has been shown that ET can be successfully treated by deep brain stimulation (DBS). This clinical treatment involves the surgical implantation of electrodes into the brain, through which current is applied. However, the mechanisms of how DBS achieves clinical benefit continue to be debated. A key question is whether ET can be modeled as a pathological network behavior as has been suggested previously. If so, we can then ask how DBS would modulate this brain activity. Our study combines: (i) simultaneous electrophysiological recordings from the brain and muscle; (ii) computational modelling; (iii) mathematical analysis. We found that the network supports oscillations in the tremor range, and the application of high frequency DBS switches this to low amplitude, high-frequency activity. We propose that our model can be used to predict DBS parameter settings that suppress pathological network activity and consequently tremor. In summary, we provide the first population level model of essential tremor including the effect of DBS on network behaviour.

## Introduction

Essential tremor (ET) is purported to be the most common movement disorder [1–4], affecting one percent of people. This disorder, which is characterised by an uncontrollable shaking of the affected limb(s) at a frequency in the range of 4-10Hz [5], is detrimental to activities of daily living [6]. While the neurophysiological underpinnings remain elusive, a number of brain regions are implicated in the underlying pathology. The thalamus has long been known to be central to if not the generation, then the maintenance of tremor, as lesioning the motor thalamus, specifically the Ventral intermediate (Vim) nucleus, leads to dampening of the tremor [7]. Interestingly, more than 50 years ago, it was reported that low frequency electrical stimulation of the thalamus reinforced tremor [8]. Furthermore, while the role of the thalamus in tremor is undisputed, for essential tremor in particular, it is the involvement of the cerebellum which differentiates it from other tremors. In particular, work has shown structural changes in the cerebellum with ET, such as neurodegeneration. Interestingly, it has been reported that ET disappears after stroke in the thalamocortical-cerebellar network [9]. In addition, disturbances of cerebellar functions, such as gait and eye blink conditioning [10] have been reported in patients with ET.

More recently, it has been shown that ET can be successfully treated by deep brain stimulation (DBS) [11]. DBS involves the surgical implantation of electrodes into disorder specific target regions, via which the neural tissue is stimulated using trains of electrical pulses. The treatment works well, with 69% of patients showing total or significant suppression of tremor (Medtronic DBS Therapy for Parkinson's Disease and Essential Tremor Clinical Summary, 2013). However, the efficacy of this method is influenced by two factors: (i) the accuracy with which the electrode is located in the affected region, and (ii) the stimulation parameter combination. While the former is typically determined using imaging prior to surgery, the scientific evidence regarding the implications of varying each DBS parameter, namely amplitude, frequency and pulse width, is relatively scarce. Thus, the latter is typically chosen by the clinician using trial and error. As such, this process of parameter determination can be time consuming and difficult, not to mention frustrating for the patient. At present, the possibility of optimising this process, by predicting stimulation parameters that would maximize the beneficial effect and minimize unwanted side effects, is limited by the lack of knowledge about the neuronal mechanisms behind either the disease itself or the therapeutic effects of DBS.

One popular hypothesis about the neurophysiological mechanisms is that tremor is caused by synchronous oscillatory activity involving thalamus, cerebellum and the motor cortex [12] and that DBS disturbs pathological synchrony. DBS also allows us to record local field potentials (LFP) from the implanted electrodes whilst simultaneously recording muscle activity (EMG). Such work has previously demonstrated that recorded LFPs contain synchronised activity at tremor and double tremor frequency [13–15]. While the thalamocortical—cerebellar network has been implicated in such activity, no modelling study has looked at whether the dynamics of this network could indeed support oscillatory activity. In this study, we therefore modelled this network and, investigated its capacity to oscillate at the frequency of synchronous activity, which we recorded from ET patients undergoing DBS surgery.

## Materials and Methods

### Ethics statement

The data was recorded as part of routine clinical practice and was stored and analysed anonymously. Informed consent was obtained from patients for the use of this data for research, and this was approved by the local research ethics committee (Charing Cross Research Ethics Committee).

### Electrophysiological recording

LFP signals were recorded from a total of 14 electrodes implanted in the motor thalamus (Vim nucleus) of seven patients with essential tremor (see Table 1). Recording techniques have been previously reported [16–18] and are summarised here: LFPs were recorded via the implanted electrodes (model 3389; Medtronic Inc., Minneapolis, MN, USA) during surgical implantation with a sampling frequency of 2 kHz. Simultaneous recordings of LFPs were made with three or four adjacent pairs of electrode contacts in a bipolar configuration. Signals were filtered between 0–1 kHz, amplified with a gain of 10,000 (CED1902, Cambridge Electronic Design, Cambridge, UK). Simultaneously, EMG was recorded via a tripolar arrangement on the affected limbs, typically from the wrist flexors. Recordings were made while the patients were awake, off any anti-tremor medication and in up to three conditions: at rest where we asked the patient not to move (n = 7), maintaining a posture by holding the arm outstretched (n = 5) and making voluntary movements such as repeatedly flexing the wrist (n = 3).

**Table 1:** Table of patient details. The patient details for the seven patients with ET who underwent DBS surgery and whose local field potential data was used in the study. We include age at time of DBS surgery, gender and tremor grading scores for right and left arms in four positions: rest, held at nose, held outstretched and whilst making a reaching movement (intention). All signals were down sampled to a sampling frequency of 64 Hz. The power spectra of the filtered LFP signals were obtained using a Fourier transform (fft function in Matlab). Cross-coherence was performed between the LFP and EMG signals (mscohere function in Matlab, Mathworks), using a periodic Hamming window, with the number of steps set to 32 and 50% overlap. Spectra were averaged across electrodes and sides of the brain and then across patients for each condition. The results obtained from this data analysis were used to constrain the behaviour of the computational model, by comparing the peak frequency of the cross-coherence to the frequency of oscillations produced by the model.

Patient number	Age at time of DBS	Gender	Tremor grade							
			Measured pre DBS (patients 1–7) or post DBS but off stimulation (patient 5 only)							
			Right				Left			
			Rest	At nose	Held out	Intention	Rest	At nose	Held out	Intention
1	70	F	0	2	3	3–4	0	2	2	3
2	51	M	0	2–3	2–3	3	0	3–5	3–4	4
3	79	M	0	4–5	8	3	0	5	7	4–5
4	67	M	5–6	5–7	5–6	4–5	4	3–4	3–4	3
5	57	F	0	0	1	2	2	2	5	5
6	49	M	0	0–2	3–4	2	0	0–1	2–3	2
7	57	M	3	4	7	3	1	2	1	3

### Computational model

**Fig 1:**
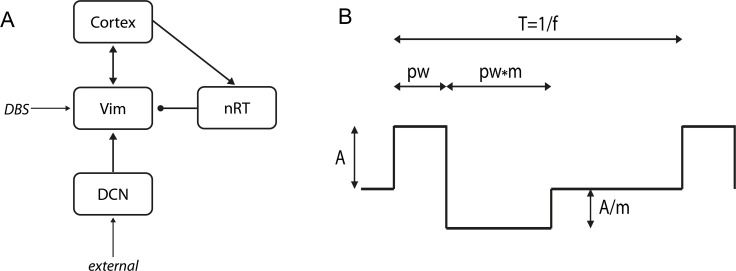
Schematic representation of the network modelled in the study. (A) The model contains four populations, the motor cortex, the Vim nucleus of the thalamus, the reticular nucleus of the thalamus (nRT) and the deep cerebellar nuclei (DCN). In addition, there are two additional inputs, a constant external input in through the cerebellar population and the DBS input into the Vim. (B) The DBS input, unlike the external input, is not tonic, but oscillatory over time. We used a biphasic square pulse to mirror the pulses used in clinical therapy. The waveform is defined by the following parameters: frequency (f), amplitude (A), pulse width (PW) and the multiple for the biphasic phase (m).

We adopt a simple population representation of the network hypothesised to underlie ET (Fig 1A). This is based on previous descriptions of the essential tremor network which include these brain regions (e.g. [12]). To model the network, namely cortex, cerebellum and thalamus (Fig 1A), we used the population-level Wilson-Cowan approach [19]. This framework has been extensively used to describe interacting populations of excitatory and inhibitory neurons [20–26]. The main assumption is that the neurons in a population are in close spatial proximity, hence the model ignores spatial interactions and deals only with temporal dynamics. The modelled variable is the proportion of cells in a population which are firing action potentials per unit time.

We specifically model two thalamic populations, the excitatory Vim nucleus and the inhibitory reticular nucleus (nRT). The latter has been implicated as a crucial player in thalamic oscillations [27–29]. In addition, we model an excitatory population of cortical neurons, representing the motor cortex (Cx) and an excitatory population of cerebellar neurons, representing the deep cerebellar nuclei (DCN), the main output of the cerebellum. Therefore, the model comprises of four first-order coupled differential equations:

$$\tau_{Vim}\frac{dE_{Vim}}{dt}=-E_{Vim}(t)+(k_{e}-E_{Vim}(t)).Z_{e}(w_{1}E_{Cx}(t)+w_{2}E_{DCN}(t)-w_{3}I_{nRT}(t))$$

$$\tau_{Cx}\frac{dE_{Cx}}{dt}=-E_{Cx}(t)+(k_{e}-E_{Cx}(t)).Z_{e}(w_{4}E_{Vim}(t))$$

$$\tau_{nRT}\frac{dI_{nRT}}{dt}=-E_{nRT}(t)+(k_{i}-I_{nRT}(t)).Z_{i}(w_{5}E_{Cx}(t))$$

$$\tau_{DCN}\frac{dE_{DCN}}{dt}=-E_{DCN}(t)+(k_{e}-E_{DCN}(t)).Z_{e}ext$$

Here, *E*_*i*_ (*i* = Vim, Cx or DCN) and *I*_*j*_ (*j* = nRT) represent the number of active neurons in the relevant excitatory or inhibitory population at a given time. The strength of the connection between two populations is given by *w*_*n*_, where *n* = 1, 2… 6. The value of this parameter is calculated by taking the product of the average number of contacts per cell and the average postsynaptic current induced in the postsynaptic cell by a presynaptic action potential. Note that the last equation, for the DCN population, is independent of the dynamics of the other three populations, and only provides an input into the Vim population. Therefore, in this model, the DCN population will tend to a stationary value and not oscillate.

The response functions *Z*_*e*_*(x)* and *Z*_*i*_*(x)* in the model represent the proportion of cells firing in a population, for a given level of average membrane potential activity *x(t)* of cells across the population. In [19], these functions were derived by assuming that the population has a distribution of neural thresholds and that all cells in the population have the same average level of membrane potential activity. Alternatively, the cells in a population can be assumed to have the same threshold but there is a distribution of the number of afferent synapses per cell. Either approach leads to the response functions being represented by monotonically increasing sigmoid function, as follows:

$$Z_{p}(x)=\frac{1}{1+exp(-b_{p}(x-\theta_{p}))}-\frac{1}{1+exp(b_{p}\theta_{p})}$$

where *p* represents *e* or *i*, *b*_*p*_ and *θ*_*p*_ are constants, and x is the level of input activity. Following Wilson & Cowan, the following values are used for these constants are: *θ*_*e*_ = 1.3, *b*_*e*_ = 4, *θ*_*i*_ = 2.0, and *b*_*i*_ = 3.7.

Therefore, this model effectively assumes that the inputs to a network are weighted, summed and thresholded.

The parameters *k*_*e*_ and *k*_*i*_ in the model are the maximum values of the response functions and are given by *k*_*e*_ = 0.9945 and *k*_*i*_ = 0.9994. Each of the parameters *τ*_*i*_ represents the time constant of the change over time in the proportion of nonrefractory cells which are firing in a population, in response to the change over time in the average membrane potential activity of the cells. The value of the time constant for each population is usually assumed to be equal to the membrane time constant of the cells in the population, as in [30] and [24]. This value is usually assumed to lie within the range 10–20 ms [30] and time constants of 10 ms were chosen as nominal values for all populations, and this parameter was left unchanged throughout the simulations.

### DBS input

The application of a high frequency input to the Vim nucleus of the thalamus was modelled using a biphasic square pulse as follows:

$$DBS(t)=A*H(\sin\left(2\pi ft\right)*(1-H(\sin\left(2\pi f(t+pw)\right))-\frac{A}{m}*H(\sin\left(2\pi f(t-pw*m)\right)*(1-H(\sin\left(2\pi ft\right))$$

where *A* is the amplitude of the input in arbitrary (arb.) units, *H* is the Heaviside function, *f* is the frequency, *pw* is the pulse width and *m* is the multiple for the charge balancing pulse (Fig 1B). This was included as an additional term into the equation for the Vim population, such that the Vim equation subsequently changed to the following:

$$\tau_{Vim}\frac{dE_{Vim}}{dt}=-E_{Vim}(t)+(k_{e}-E_{Vim}(t)).Z_{e}(w_{1}E_{Cx}(t)+w_{2}E_{DCN}(t)-w_{3}I_{nRT}(t)+DBS(t))$$

### Bifurcation analysis

To further analyse any oscillatory behaviour displayed by the modelled network, we used bifurcation analysis of our four equations using a numerical analysis approach in a software package called LOCBIF [31]. For a system of ordinary differential equations, the nature of the fixed points of the system can change as the parameters are varied. If a fixed point changes stability, appears or disappears we say that a bifurcation has occurred. At points of bifurcation the behaviour of a system changes in a way that depends upon which type of bifurcation has happened.

In this study, bifurcation analysis revealed that an oscillation can arise through an Andronov–Hopf bifurcation or a SNIC bifurcation (saddle-node on an invariant curve). At the point of an Andronov–Hopf bifurcation, a stable equilibrium point loses stability and a particular type of trajectory can appear in the neighbourhood of the fixed point. This trajectory is called a limit cycle, which is an isolated closed curve. Motion of the system along the limit cycle trajectory is periodic and hence oscillatory behaviour is encountered (see for example [32] for more details).

This limit cycle behaviour is illustrated in bifurcation diagrams for the model parameters. The curves shown in these diagrams indicate points in the parameter space where a bifurcation occurs and a limit cycle arises. Thus the curves separate the portion of the parameter space where the system has a fixed stable equilibrium point from the portion where a limit-cycle oscillation is present. These diagrams are produced in each case by keeping all but two of the parameters at their control values.

## Results

### LFP recordings display tremor band activity

**Fig 2:**
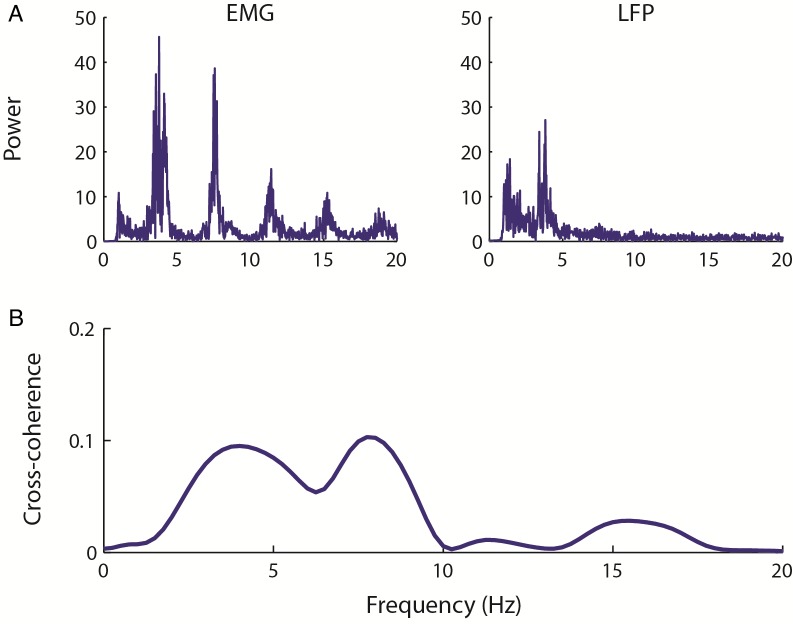
A representative simultaneous recording of EMG and LFP from one ET patient. In (A), the power spectra are shown for the EMG and the LFP across the entire recording. The EMG spectrum clearly shows a tremor band peak at 4Hz and subsequent peaks at the harmonic frequencies. The LFP also shows a 4Hz peak, although with smaller power. (B) The Cross-coherence between the EMG and LFP reveals a peak at 4Hz and the harmonic frequencies.

DBS patients routinely have intraoperative recordings made while they have the electrodes inserted into the thalamus. This allows us to measure the neural activity patterns associated with ET and can further aid the targeting of the thalamus by locating neural patterns associated with the thalamus. Fig 2 shows an example recording from a single patient. Fig 2A shows the power spectra for a single EMG (left) channel and one LFP (right) channel from the contralateral hemisphere. The data shows that there are clear peaks in the EMG power spectra at approximately 4 Hz and at the harmonic frequencies of 8 Hz, 12 and 16 Hz. In the LFP power spectra, a peak at tremor frequency is also seen around 4Hz, and an indication of an increase in power at 8Hz. The cross-coherence between the EMG and LFP signals is shown in Fig 2B, and clearly shows a peak at tremor frequency, double tremor frequency and somewhat at the subsequent harmonic frequencies.

**Fig 3:**
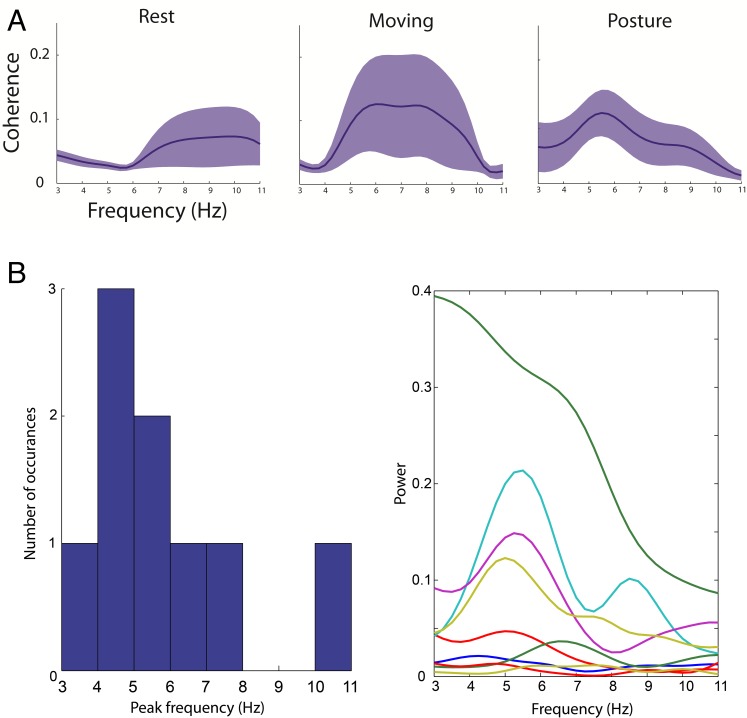
The EMG and LFP data over epochs. (A) The data for each of the patients was first split into different behavioural epochs: rest, when the patient was asked to refrain from moving (seven patients, 14 spectra); moving, when the patient was asked to make self-paced repetitive movements such as opening and closing the hand (five patients, nine spectra); and posture, when the patient was asked to hold their arm out (three patients, four spectra). We can see that in all epochs there is an increase in tremor-band coherence, with a more tuned increase in the moving and posture conditions. (B) In addition to the mean cross coherence, in order to further demonstrate the variability across patients, we show the cross-coherence for the posture epoch for all nine spectra and a histogram summarising the peaks of the cross-coherence spectra in the 3–11 Hz range.

We split our data into the three different behavioural epochs: rest, self-paced voluntary movement and maintaining a posture. We averaged the data for each of our seven patients across channels for the two hemispheres, and Fig 3A shows the averaged EMG-LFP cross coherence across patients for each epoch (shading indicates between subject SEM). In all conditions, there is increased cross-coherence within the tremor frequency band. In the rest condition, patients were instructed not to move, but the observed increase in 6–11 Hz cross-coherence may be related to movement and/or rest tremor. When moving (hand is repeatedly open and closed) or maintaining a posture (arm is held up with the hand by the nose) the cross-coherence shows a more tightly tuned, specific increase at 5–9 or 5–6 Hz depending on the epoch. Furthermore, the individual cross-coherence spectra and the histogram of peak frequency in these spectra (Fig 3B) in the 3–11 Hz range clearly demonstrate that there is variability amongst the patients and recordings, which may reflect a number of individual differences, including the precise electrode location and differences in the performed motor behaviour.

### Network supports tremor band oscillations

**Fig 4:**
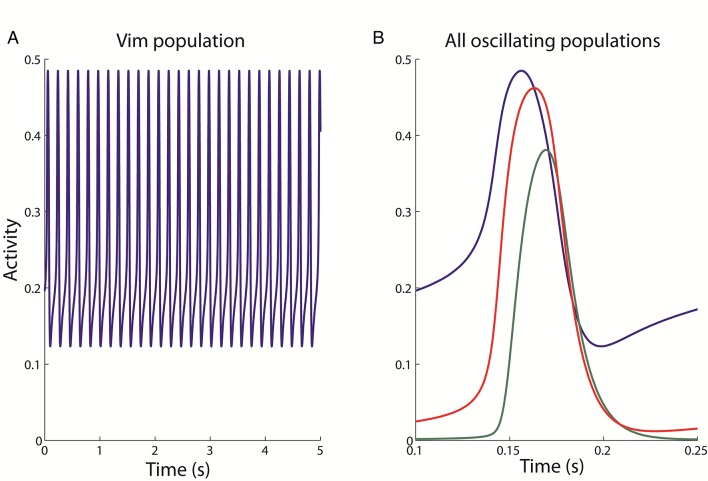
The oscillatory activity observed in the model. The activity of the Vim population alone (A) is shown over 5 seconds of simulated time, and for the three populations (B) displaying oscillations for approximately one cycle (0.25 seconds). The oscillation amplitude is stable over time for any one population, but varies across populations. The oscillation is lead by the Vim population (blue), then followed by the nRT (red) and finally the cortex (green).

In order to investigate the dynamics of the network suspected to be responsible or necessary for such oscillatory activity, we constructed a population model of the thalamocortical-cerebellar network as described above. This network was simulated by exploring the connection weights parameter space to uncover regions which produced oscillatory activity in the typical tremor frequency range, as observed in our EMG-LFP data. We found that the network readily oscillated at a frequency of 5.5Hz, when the weights were set at the values given in Table 2. Given these baseline weights, all of the neuronal populations in the network, except the DCN, oscillate at this frequency (the Vim oscillations are shown in Fig 4A). The Vim leads the cortical oscillation by 6.8 ms (Fig 4B), which is consistent with previously measured lags [33]. This baseline oscillatory activity was examined in more detail using bifurcation analysis.

**Table 2:** Model parameters. The free parameters used in the Wilson-Cowan model of the thalamocortical-cerebellar network for essential tremor. The weight parameters for the six connections and the time constants in the model network are given here, and all other parameters are listed in the text. These parameters were selected prior to the bifurcation analysis, by sweeping the parameter space and were chosen to be close to the bifurcation point where the oscillation exists with the correct frequency. The *w* symbols are those appearing in the four equations describing the network in the methods section.

Connection			Weight
From	To	w	
**Cortex**	**Vim**	***w4***	**12**
**DCN**	**Vim**	***w1***	**6**
**nRT**	**Vim**	***w2***	**12**
**Vim**	**Cortex**	***w3***	**10**
**Cortex**	**nRT**	***w5***	**10**
**Time constant**		**τ**	**10 ms**

### Bifurcation analysis demonstrates importance of connections

**Fig 5:**
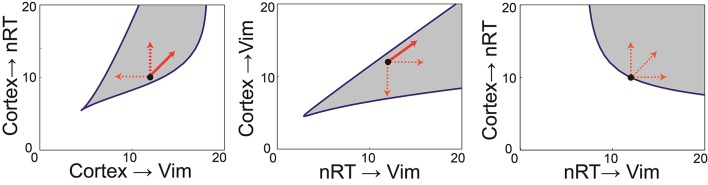
Bifurcation analysis of the connection weights. The relationship between the six connection weights can be examined by using bifurcation analysis, which allows us to co-vary any two parameters at a time and trace out the region in parameter space where the bifurcation leading to oscillatory activity occurs. In this way, we can split the parameter space into oscillatory (shaded) and non-oscillatory regions and therefore make predictions about the network structure in the pathological state. Furthermore, we examined the frequency of the oscillations throughout these shaded regions and found that the frequency of oscillations does not remain constant. In fact, as parameters varied from the default values (black circle), the frequency increased (solid line) or decreased (dashed line) as shown in the plots. The frequency of oscillations we observed within these regions however, was between 2 Hz and 8 Hz.

Once established, we did not change any of the model parameters from those in Table 2 when subsequently applying DBS stimulation to our network. However, before doing so, we wanted to investigate the relative contribution of the connections within the network on the oscillatory activity we observed. We used bifurcation analysis to locate stable points in our parameter space and vary two parameters at a time while observing the stability of the system. All but two parameter values at a time were fixed (as in Table 2) for the determination of each bifurcation curve. We used specialised software (LOCBIF) for the bifurcation analysis to find a region in the 2D plane of the two selected parameters corresponding to the oscillations. We found that the observed tremor frequency oscillation arises in our model through an Andronov–Hopf bifurcation nearby of which a limit cycle is observed. We plotted bifurcation diagrams for pairs of weight parameters in the model which kept the model at this bifurcation point, and these are shown in Fig 5. The shaded regions indicate the region of parameter space for which the model will display oscillations, although the frequency of the oscillations does not remain constant throughout these shaded regions, as described below.

This analysis revealed the following features about the importance of connection weights relative to one another. First, Fig 5A shows that the cortical input to the Vim and the cortical input to the nRT must be balanced in order to maintain oscillations, as the oscillatory space lies along the diagonal. Furthermore, if the cortical drive to the Vim decreases, the frequency of oscillations decreases, but if the cortical input to the nRT increases, or both weights increase together, the frequency of oscillations increases. Second, the reticular input to the Vim should on the whole be stronger than the cortical input (shaded region is mainly below the line of equality in Fig 5B). Interestingly, if those inputs both increase in weight, the frequency increases, but a move to any other region of the oscillatory space results in a decrease in frequency. Third, the inhibitory loop (cortex to nRT and nRT to Vim) must be maintained with weights no less than the default values for oscillations to be present. All other regions of the oscillatory parameter space result in lower frequency oscillations. Finally, one of the weight parameters (cortical input to Vim) could be set to zero with oscillations maintained in the network, but only with a corresponding increase in cerebellar drive. Hence no population (and its corresponding connections) could be lost and oscillations maintained, that is all of the populations are critical to oscillatory behaviour.

### DBS has differential effects at different amplitudes and frequencies in the modelled network

Given that our model network was able to oscillate at tremor frequency, the next step was to see how this behaviour changed when an input mimicking the effects of DBS was applied to the thalamus. In the following, we will call the oscillatory network activity before DBS application the baseline oscillation. We applied a biphasic square pulse at different amplitudes and frequencies into the Vim population of the model network only, to replicate targeted DBS of the thalamus. We found two effects of DBS on the network activity that we discuss in turn.

**Fig 6:**
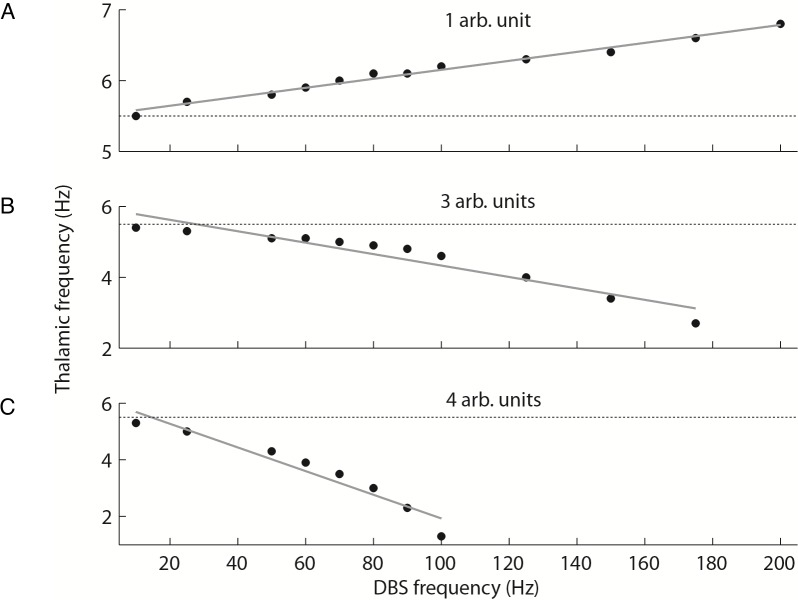
DBS effects on oscillatory activity. When the DBS input is applied to the Vim, the effect on the oscillatory activity is both amplitude and frequency dependent. (A) At low amplitudes, DBS changes the frequency of the oscillation, and the relationship between the applied and the resulting frequency is linear. (B) At higher DBS amplitudes, this relationship changes, and the higher the DBS frequency the lower the frequency of the high amplitude oscillation, which is eventually replaced by the low-amplitude, high frequency activity. When the DBS amplitude increases further (C), this switch from low-frequency, high-amplitude activity to low-amplitude, high-frequency activity occurs at a lower stimulation frequency.

First, DBS at low amplitudes altered the baseline oscillatory activity of the network, not in amplitude, but frequency. Fig 6 shows this relationship quantitatively for three different DBS amplitudes. At an amplitude of 1 arb. unit, DBS increased the frequency of the oscillatory activity at all stimulus frequencies greater than 10 Hz. The relationship between stimulation frequency and thalamic frequency was found to be linear (r^2^ = 0.98) up to a DBS frequency of 200Hz, beyond which the thalamic frequency plateaued at a maximum 6.8 Hz. It is interesting to note that this linear relationship changed when the stimulation amplitude was increased to 3 arb. units. At this point, the DBS frequency was inversely related to the thalamic frequency (r^2^ = 0.91), with higher DBS frequencies slowing the underlying oscillatory activity until it was completely suppressed. For the 3 arb. units stimulation, this suppression occurred at frequencies greater than 175 Hz. Similarly, at an amplitude of 4 arb. units there was an inverse relationship between DBS and thalamic frequency (r^2^ = 0.93). The slope of the linear fit to the data was much steeper in this case, and the oscillations were suppressed at frequencies greater than 100 Hz. This trend was observed for increasing DBS amplitudes, such that as the amplitude increased, the frequency at which DBS would suppress the thalamic oscillations decreased. However, the limit was that for DBS frequencies less than or equal to 25Hz, even extremely large amplitudes (100 arb. units) did not suppress the thalamic oscillations.

Second, DBS induced a switch from large amplitude low frequency baseline activity to small amplitude high frequency activity. For a fixed value of the stimulus frequency, as we increase the DBS amplitude there is a critical point which eliminates the baseline oscillation and induces the switch to high frequency network activity. Thus, if DBS amplitude is higher than this threshold then the baseline oscillation disappears and high frequency, low amplitude activity becomes the dominating neuronal activity mode.

**Fig 7:**
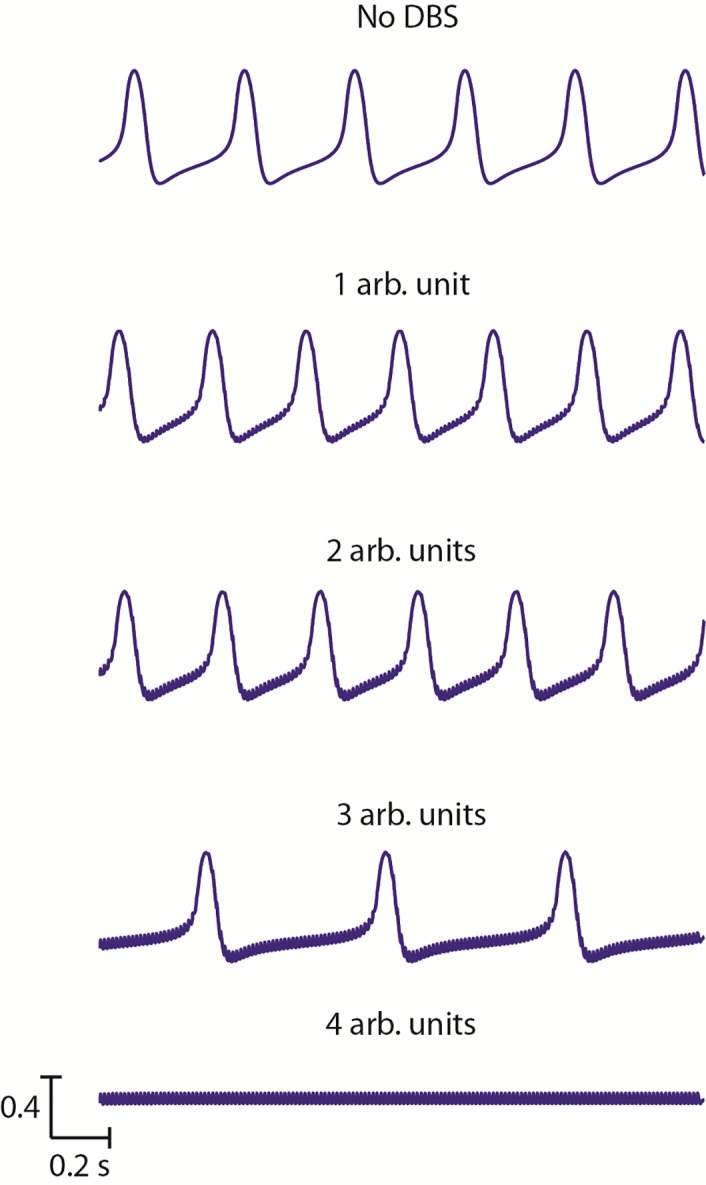
The change in oscillations with increasing DBS amplitude. For a single DBS frequency, the change in Vim activity is shown as the amplitude of the stimulation increases. The baseline large amplitude tremor band activity is shown at the top, and the low-amplitude high frequency activity at the bottom. In between, the activity gradually switches from one to the other, with an initial increase in frequency at low amplitudes, followed by a decrease in frequency as the amplitude increases further.

Fig 7 shows both of the effects described above, the change in the baseline oscillation frequency and the switch to high frequency activity for a DBS stimulation frequency of 150 Hz. In Fig 7, at 1–3 arb. units of DBS amplitude, the network activity remained low frequency and high amplitude, but the frequency decreased compared to the baseline (no DBS) condition. However, at 4 arb. units, this activity was abolished and there was a switch to high frequency, low amplitude activity. For all DBS frequencies between 70 Hz and 100 Hz, this switch occurred at 5 arb. units and for frequencies greater than 100 Hz this occurred at 4 arb. units. That is, DBS drove the thalamic activity at stimulation frequency up to a maximum of 167 Hz, and beyond this point the network could no longer follow the stimulus frequency. Applying DBS at different amplitudes and at therapeutic frequency or at different sub-optimal frequencies lead to the underlying oscillations changing frequency or being replaced by irregular high frequency activity depending on the stimulus amplitude.

**Fig 8:**
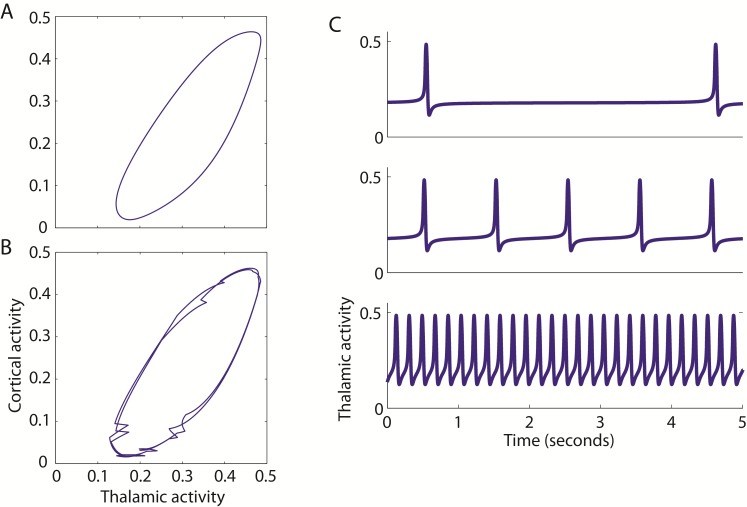
The SNIC bifurcation. The baseline oscillation in the network is described as a limit cycle, which can be seen when the activities of two populations (Vim and cortex) are plotted against one another over time (A). When the DBS input is applied to the network, the limit cycle is no longer constant and deviates from a perfect cycle over time (B). This is caused by a saddle-node on an invariant circle (SNIC) bifurcation, which can be seen here when the ext parameter value is gradually increased to its value in Table 2 (C).

Interestingly, in the presence of the DBS stimulus, we found another bifurcation near the Andronov-Hopf bifurcation. A limit cycle is observed near an Andronov-Hopf bifurcation, and can be seen as a cyclical relationship between two of the time dependent variables in the model equations. An example of the limit cycle in the absence of DBS is shown in Fig 8A, where the thalamic and cortical activity are plotted as a function of one another. However, when DBS is applied, the limit cycle is not constant over time, but varies (Fig 8B). Furthermore, the presence of this saddle-node on an invariant circle (SNIC) bifurcation is confirmed in the absence of DBS, when the frequency of the baseline oscillation can be decreased by altering the value of the ext parameter (Fig 8C).

## Discussion

The thalamus has been known to be involved in oscillatory activity such as spindling [27–29]. In this study, we hypothesised that the dynamics of the thalamocortical-cerebellar network would be able to support the pathological synchronous activity, which has been thought to be the signature, if not the cause, of essential tremor [12, 34]. Such oscillatory activity has been previously recorded not only in the muscle activity via EMG, but also in the brain via EEG, LFP and microelectrode recordings [13, 14, 35]. Here we tested our hypothesis, by examining the population level dynamics of the network constructed using a Wilson-Cowan approach [19]. We found that this network does indeed oscillate readily in the essential tremor frequency range, also reflecting the activity we recorded in patients with essential tremor undergoing DBS surgery.

Our model demonstrates that the dynamics of this thalamocortical-cerebellar network supports oscillatory activity in the tremor range. Previous computational modelling work examining the effects of DBS has mainly been directed to studying Parkinson’s disease and either focussed on unconnected neurons [36–39], axons [40–43], or local networks [44] made up of conductance-based neurons. The pathophysiological state in these two diseases may have some similarities, and this study indicates that such activity is not only reliant on the biophysical properties of the neurons, but emerges from the structure of the tremor network itself, which involves different brain regions in Parkinson’s disease and Essential tremor. The thalamus has been implicated in various types of oscillatory activity [27–29] [25, 26] [45–47], our results are consistent with such views of the thalamus and interestingly, we find here that though the corticothalamic connection is important, it is the driving input from cerebellum which needs to be the strongest input into Vim.

The oscillations in this network arise from an Andronov-Hopf bifurcation. Therefore close to the point of bifurcation, there will be small amplitude oscillations and the frequency of these oscillations will be fixed. As we move away from the bifurcation point, the amplitude and indeed frequency of the oscillations can vary. Furthermore, bifurcation analysis of the network showed that the oscillatory activity is robust under parameter variations, while important relationships between the connection weights exist. In particular, we see that quantitative relationships between connection strengths must be maintained for the pathological oscillations to be sustained. For example, we found that all of the populations in the network are critical to oscillatory behaviour. This is consistent with reports of patients with ET, who have seen improvement in their tremor following a stroke in the thalamocortical-cerebellar network [9]. We also predict that the driving input to the Vim from cerebellum must outweigh the cortical feedback to maintain oscillations. This prediction is in agreement with the hypothesis that ET occurs due to a lack of GABA in the cerebellum, which in turn leads to disinhibition [48, 49].

Such predictions about the state of the network in disease can be further tested experimentally either in vitro or in vivo animal work, or in human work via imaging. For example, the harmaline animal model of ET, the most studied model, suggests that tremor emerges due to rhythmic inferior olive firing [50]. This model further suggests that DCN, which is part of the network modelled here, is involved in harmaline tremor as c-Fos expression is induced in the DCN as a result of the manipulation [51]. While the precise role of the DCN is unclear, the lesioning of the DCN can lead to action tremor in humans and primates [52]. High frequency stimulation of the thalamus in mice, has been shown to suppress harmaline tremor, indicating that a similar network to ET, may indeed be involved [53]. Therefore, such animal models may be used to test our predictions about the relative strength of connections between brain regions. DTI imaging of patients with ET, which until now has been limited and produced conflicting results, could also be used to further elucidate the network structure of ET. A recent review [54] indicates a difference between ET and control participants predominantly in the cerebellar peduncles.

In particular, we studied the impact of a DBS like input at therapeutic frequency on our network activity. We found that such an input suppresses the oscillations and drives a higher frequency, low-amplitude activity across the network. This is consistent with previous modelling work [55, 56] using conductance-based model neurons, indicating that the use of network models which are relatively easy to set up, analyse and relate to data is another valid methodology for studying DBS. It is important to note however, that in our model the application of DBS which is applied to the Vim nucleus alone, will affect all efferent connections of the Vim. Our model does not account for antidromic activation, as the connections are unidirectional. In future work, we could explicitly model the afferent connections, but in that case these connections would participate in the network activity even in the absence of DBS, and would change the entire dynamics of the model. Furthermore, this effect reflects a hypothesis about subthalamic DBS, which proposes that it acts to replace pathological synchrony with low amplitude activity and regain information flow through the thalamus [57–59]. Interestingly, while this hypothesis has been proposed for subthalamic DBS to treat Parkinson’s disease, our results indicate that a similar mechanism may apply for thalamic DBS for ET. Another interesting observation is that the high frequency activity which was driven by DBS, matched the DBS frequency but only up to a maximum frequency of 200Hz, we predict that a similar result would be seen in single neuron simulations or recordings.

The model presented here also showed frequency dependent effects with DBS-like stimulation. While previous work has examined the impact of low or high frequency DBS [61, 66], there has been no in vivo study reporting the effect of systematically varying the DBS frequency, either on the clinical symptoms or electrophysiological recordings. Consequently, our model allows these experiments to be done in silica. We found that at low frequencies, DBS did not abolish the tremor band oscillatory activity as readily as at higher frequencies. In fact, the lower frequency stimuli maintained the low frequency, high amplitude activity for a wider range of stimulus amplitudes. This effect may be linked to the clinical observation that for treating essential tremor, high frequencies are necessary [60] Interestingly, for frequencies greater than 30 Hz, we were able to abolish the tremor band activity in the network if the amplitude of DBS was increase, which has also been shown previously [61]. It was shown more than 50 years ago that low frequency stimulation of the thalamus strengthen tremor, in patients undergoing stereotactic surgery [8]. This predicts that such low frequency stimulation, may not necessarily drive tremor, but allow the network to sustain the underlying pathological oscillation, rather than suppress it as with higher stimulation frequencies. Recently, it has been discussed whether uniform regular stimulation or patterned stimulation such as repeated bursts of high frequency stimulation is most effective [62]. We applied bursting stimulation patterns in this model but found it to be less effective at suppressing the pathological oscillation in this model in agreement with previous work [63].

Furthermore, it has previously been observed clinically and recently shown theoretically that kilohertz frequency stimulation is also effective at suppressing tremor, but only up to a limit of approximately 3 kHz [61, 64]. In our model, we found that stimulation up to 3.5 kHz was still effective at suppressing the low frequency, high amplitude oscillations. The model also showed amplitude dependent effects of DBS. At low amplitudes, DBS increased the tremor band activity in a linear fashion, while at higher amplitudes DBS decreased the tremor band activity with an inversely linear relationship. A physiological study has previously reported an increase in tremor frequency with DBS as we observed at low amplitudes {Vaillancourt, 2003 #2396]. However, in that study the increase was independent of DBS amplitude. These differences in the frequency and amplitude dependent effects may highlight the limitation of such network level dynamic models, that they do not capture the full spectrum of neuronal dynamics. It is critical to note, that the Wilson-Cowan model however, does not account for the detailed firing properties of neurons, such as the distinction between burst and tonic firing, a critical feature of thalamic cells that has been shown to be important in ET. Instead the Wilson-Cowan representation only allows for the firing rate of a population. Consequently, this study therefore can only look at tremor as a network phenomenon and thus the impact of DBS on that activity.

Similarly, the model presented here does not account for the spatial dimension within a population. While this is in fact an advantage of this modelling approach, we could represent different populations within a brain region, for example to represent somatotopy within the Vim nucleus [65]. Furthermore, the modelled network in our current work, only accounts for four populations. One brain region which is missing is the inferior olive, which is implicated in the pathogenesis of ET, particularly due to the work with the harmaline rodent model [50]. Further work could expand the model to include at least this important input to the cerebellum. Finally, in the current study we have used a computational model to replicate the data recorded from DBS patients. One clear extension to this parallel approach would be to fit the model parameters, particularly the connection weights, to the patient data. The aim would be to fit the parameters of the model to the peak frequency of the individual patient’s data. Furthermore, such patient specific models could also be used to simulate specific parameter settings tried in individual patients to correlate network changes to therapeutic effects or the emergence of side effects. In this way we could explore whether we could represent individual patients, who may show variations in the frequency of their tremor, with a patient specific model, not of the network structure, but of the relative strength of parameters across the network.

## Conclusions

In conclusion, we have shown that the dynamics of a network of multiple brain regions thought to be involved in essential tremor are able to support oscillations in the tremor-band frequency range, as seen in LFP recordings in patients. In addition, we have shown that the application of a biphasic square pulse into the Vim nucleus disrupts this synchronous activity. The network displays frequency-dependent behaviour which may be linked to clinical observations and makes predictions about the relative strength of connections between brain regions. This may explain one mechanism by which thalamic DBS achieves suppression of tremor in ET patients.

## Supporting Information

S1 Data The electrophysiological data used to constrain our model. (RAR)
